# Larval density in the invasive *Drosophila suzukii*: Immediate and delayed effects on life‐history traits

**DOI:** 10.1002/ece3.10433

**Published:** 2023-08-25

**Authors:** Alicia Reyes‐Ramírez, Zaïnab Belgaidi, Patricia Gibert, Thomas Pommier, Aurélie Siberchicot, Laurence Mouton, Emmanuel Desouhant

**Affiliations:** ^1^ UMR 5558, Laboratoire de Biométrie et Biologie Evolutive, CNRS, VetAgro Sup, Université de Lyon Université Claude Bernard Lyon 1 Villeurbanne Cedex France; ^2^ UMR 1418, Laboratoire d'Ecologie Microbienne, INRAE, CNRS, VetAgro Sup Université Claude Bernard Lyon 1 Villeurbanne Cedex France

**Keywords:** crowding, *Drosophila*, life‐history traits, trade‐off

## Abstract

The effects of density are key in determining population dynamics, since they can positively or negatively affect the fitness of individuals. These effects have great relevance for polyphagous insects for which immature stages develop within a single site of finite feeding resources. *Drosophila suzukii* is a crop pest that induces severe economic losses for agricultural production; however, little is known about the effects of density on its life‐history traits. In the present study, we (i) investigated the egg distribution resulting from females' egg‐laying strategy and (ii) tested the immediate (on immatures) and delayed (on adults) effects of larval density on emergence rate, development time, potential fecundity, and adult size. The density used varied in a range between 1 and 50 larvae. We showed that 44.27% of the blueberries used for the oviposition assay contained between 1 and 11 eggs in aggregates. The high experimental density (50 larvae) has no immediate effect in the emergence rate but has effect on larval developmental time. This trait was involved in a trade‐off with adult life‐history traits: The time of larval development was reduced as larval density increased, but smaller and less fertile females were produced. Our results clearly highlight the consequences of larval crowding on the juveniles and adults of this fly.

## Graphical abstract

The immediate and delayed effects of density are key to population dynamics. Our results support the existence of trade‐off between larvae and adult life‐history traits in the pest *D. suzukii*.

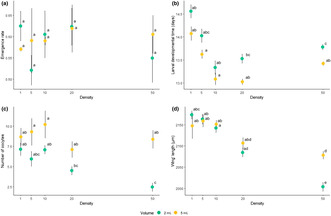

## 1 INTRODUCTION

Density dependence is a key driver of demographic parameters (Hixon & Johnson, 2009) that may have positive or negative effects on population growth rate (Mueller, 1997). These effects on demographic parameters emerge from the combination of effects occurring at lower levels of biological organization (Mueller, 1997; Ponton & Morimoto, 2020). At the individual level, we can distinguish immediate and delayed effects. Immediate effects occur at immature stages on larval traits and are associated with resource acquisition (Dethier, 1959; Putman, 1977). For many insect species, nutritional resources may be in limited quantity when females lay their eggs in a finite volume such as fruits or seeds and when the whole immature development occurs in a given seed/fruit. Therefore, female oviposition strategies affect early developmental conditions and thus larval fate as well as adult traits (Nestel et al., 2016). Delayed effects emerge in response to density‐dependence conditions during development and are expressed on adult traits (Agnew et al., 2002).

Both immediate and delayed effects can positively or negatively affect individual fitness (Peters, 2003) by altering life‐history traits and promoting trade‐offs (Parker & Gilbert, 2018). These effects are well illustrated in *Drosophila melanogaster*. Indeed, ephemeral habitats (e.g., rotten fruits) in which *D. melanogaster* larvae inhabit were usually subject to high crowding resulting in a reduction of adult size (Atkinson, 1979; Miller & Thomas, 1958). For example, the weight was reduced by up to 63% at high larval density conditions (Miller & Thomas, 1958). In *D. melanogaster*, larval and adult survival (Lewontin, 1955; Lints, 1963; Sang, 1949; Scheiring et al., 1984), and fecundity (Chiang & Hodson, 1950; Lints & Lints, 1969; Prout & McChesney, 1985) were also reduced when larval density increases. The high values of larval density were also associated with rapid development (Nunney, 1990; Partridge & Fowler, 1992) that may have different consequences on life‐history traits. A short development time was, for instance, positively correlated with reduced lifespan and adult weight in *D. melanogaster* (Prasad et al., 2000, 2001), but the opposite pattern was observed in populations of *D. ananassae* and *D. nasuta* (Sarangi et al., 2016). The differences may be due to the variability in the volume of food provided during the experiments, suggesting that fitness in crowded environments depends not only on larval density but also on the amount of resources available (Klepsatel et al., 2018; Sarangi et al., 2016). Even if usually individuals that develop in low‐density conditions are expected to have adaptive advantages in later life due to low levels of competition for resources (the “silver spoon” effect; Angell et al., 2020; Grafen, 1988), the examples of *D. ananassae* and *D. nasuta* showed that high density may also be beneficial. This is also the case in individuals expressing a greater performance in defense against predators (e.g., in the bark beetle *Ips pini*, Aukema & Raffa, 2004), in cooperative feeding (e.g., in the butterfly *Chlosyne janais*, Denno & Benrey, 1997), or when beneficial microbiota are horizontally transmitted (e.g., in the mosquito *Aedes aegypti*, Correa et al., 2018).

These impacts on life‐history traits highlight the need to investigate the immediate and delayed effects of density to predict population dynamics, especially for pest species (Alkema et al., 2019). In this study, we focus on *Drosophila suzukii* (Matsumura, 1931), a pest of many berry and stone fruit crops in Asia, Europe, and America (Dos Santos et al., 2017; Lee et al., 2011). Females possess a serrated ovipositor (Atallah et al., 2014) that allows them to lay eggs in healthy fruits, unlike most other Drosophilidae that oviposit on ripe or damaged fruits (Mitsui et al., 2006). This polyphagous fly thus induces severe economic losses for agricultural production (Knapp et al., 2021). Despite its main agricultural impacts, the immediate and delayed effects of density on life‐history traits have not been deeply investigated. Thus, few publications indicate that at high larval densities, adult weight (Kienzle et al., 2020), and survival (Wang et al., 2019) decrease. However, the magnitude of this effect can be mitigated by the quality of the diet (Hardin et al., 2015), which depends on the fruit (Bellamy et al., 2013; Hamby et al., 2016; Jaramillo et al., 2015; Shu et al., 2022; Tochen et al., 2014). A high density can also alter the chemical composition and microbial diversity of the food medium in which larvae developed due to foraging and excretion of conspecifics (Henry et al., 2020).

Here, we aimed at characterizing the immediate and delayed effects of larval density on major life‐history traits of *D. suzukii*. To fulfill this objective, we first investigated how females distribute their eggs in fruits in order to establish a relevant range of larval density per fruit. Then, we experimentally tested the effect of larval densities on larval and imaginal life‐history traits. Based on findings in other *Drosophila* species, we expected that larval crowding results in a trade‐off between developmental time and other traits and, more precisely, that individuals develop faster but have a lower emergence rate, potential fecundity, and adult size. We predicted that these changes would be more severe when resources for larvae were more limited.

## 2 MATERIALS AND METHODS

### 2.1 *Drosophila suzukii* line and rearing conditions

We used a *Wolbachia*‐free line of *D. suzukii* originated from the Agricultural Entomology Unit of the Edmund Mach Foundation in San Michele All'Adige, Trento Province, Italy (Nikolouli et al., 2020). Before and after the experiments, the absence of *Wolbachia* was checked by PCR (see Table S1 for protocol). The flies were reared on a cornmeal diet containing: 0.9% agar, 5% sugar, 3.3% cornmeal, 1.7% dried yeast, and 0.4% nipagine and maintained in an incubator at constant temperature (22.5°C) and humidity (RH = 60%) with a 12‐h light/dark cycle.

### 2.2 Oviposition assays: What range for larval density in a fruit?

The objective of this experiment was to provide an estimation of the range of larval density per fruit. Egg‐laying behavior of individual females was observed on blueberries. In a plexiglass box (23.8 × 17.8 × 2 cm), three groups of two blueberries (from FRUITS ROUGES & Co. organic farm) were placed at equal distance (18 cm) from each other (Figure S1). A piece of sugar agar medium was placed in the center of the box to ensure the nutrition of the flies. In each box, one 7‐day‐old mated female was placed for 18 h (32 replicates were done), and then, the number of eggs per fruit was counted under a binocular loupe.

### 2.3 Experimental protocol for immediate and delayed effects of larval density on life‐history traits

In order to test the effects of larval density on life‐history traits, we experimentally placed different densities of larvae (1, 5, 10, 20 or 50 larvae) in 25‐mL tubes (Eppendorf® Conical Tubes) and gave them two different volumes of resource (2 or 5 mL of cornmeal diet). The two volumes of food mimicked a large and small fruit size.

**FIGURE 1:**
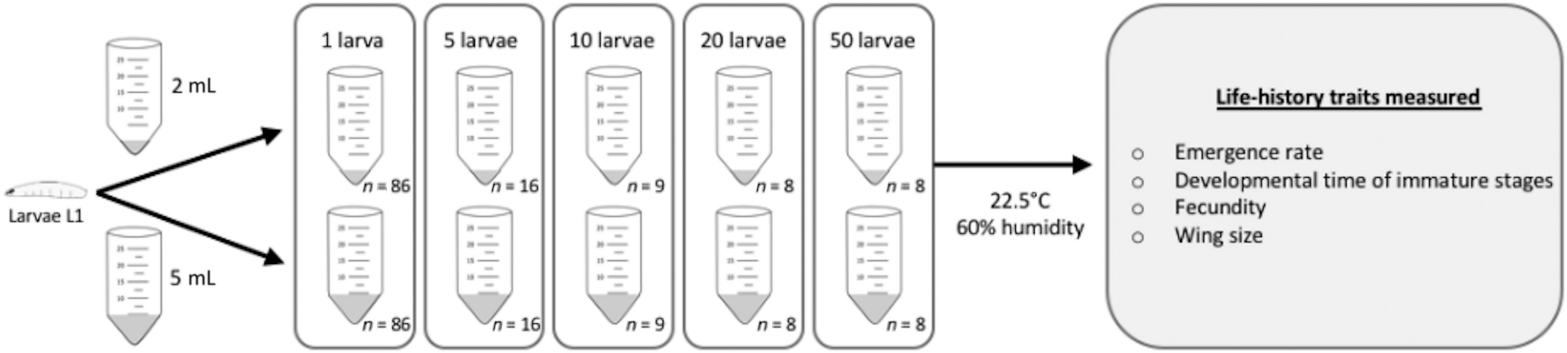
Experimental protocol for testing immediate and delayed effects of larval density on life‐history traits.

To obtain larvae, we first let a group of mated females (at least one‐week‐old) to oviposit in cornmeal diet for 24 h under standardized conditions (the same ones that were used for the flies rearing). After egg hatching, larvae of the first stage (L1) were collected and randomly assigned to tubes belonging to one of the 10 experimental modalities (5 larval densities × 2 volumes of food). For each modality, at least eight replicates (i.e., tubes) were performed (Figure 1). For convenience and due to the huge number of L1 larvae used, the experiments were conducted in two consecutive temporal blocks (1 week between the blocks).

### 2.4 Several larval and adult life‐history traits were measured

#### 2.4.1 Preimaginal developmental time and adult emergence rate

Tubes containing different larval densities and different volumes of diet were checked twice a day in order to detect adult emergence. For each tube, the emergence rate was calculated by the ratio of the number of adult flies over the number of L1 larvae added. Developmental time was estimated for each individual by the time between the date of introduction of L1 larvae into the tubes and the date of emergence.

#### 2.4.2 Potential fecundity

Individuals were sexed as soon as they emerged and placed individually in tubes (25 mL). Potential fecundity was assessed for 3‐day‐old virgin females randomly taken (at least 20). The dissection of their abdomen in PBS was performed after placing them in the freezer (−22°C) for 30 min. The number of mature oocytes was counted in the two ovaries with a binocular loupe as described in Plantamp et al. (2017).

#### 2.4.3 Wing length and width as a proxy of adult size

Prior to dissection, the right wing of females was taken and placed on a microscope slide in order to measure its size (a classical proxy of the adults' size in *Drosophila* species: David et al., 1994). Images of the wings were acquired using the AxioVisio 4.8 software on a Zeiss Imager.Z1. microscope. Two measures were performed per modality: The length corresponds to the distance between the tip of the wing and the R_4 + 5_ vein, and width to the distance between the R_2 + 3_ and the Cu_A1_ veins (see Figure S2; Debat et al., 2008; Stockton et al., 2020; Tran et al., 2020).

### 2.5 Statistical analysis

To test whether, after 18 h of oviposition period, the egg distribution was aggregated or random, we fitted different theoretical distributions (GLMs with Poisson, negative binomial (NB), and zero‐inflated negative binomial (ZINB) distributions, with log and logit link, respectively) to the number of eggs deposited per fruit and to the number of fruits infested per female. NB and ZINB are usually used to fit aggregated distributions. ZINB is used for overdispersed count data, allowing to model data with excessive zeros. The AIC values were used to compare the different fitted models. We tested whether there was a differential oviposition rate between females by the means of a generalized linear mixed model (GLMM) adjusted with a Poisson distribution. The total number of eggs was the dependent variable, while the box (i.e., the female) was the independent variable. The date on which the replicates were made and the group of blueberries' pairs were included as a random factor.

The effects of larval density on emergence rate (i.e., the total number of emerging adults per tube according to the initial number of L1 larvae) were analyzed with a GLM (binomial distribution and logit link). We included the volume of resource (categorical variable), the density (continuous variable), the density square, and the double interactions as independent variables. The density square was added to test for nonlinear relationships between density and the life‐history traits.

We fitted different GLM to analyze development time (GLM with Gamma distribution), potential fecundity (GLM with Poisson distribution), and size of the wings (length and width, GLM with Gaussian distribution). For each model volume, density, density square, and the double interactions were the independent variables. In the case of development time, the sex of the emerging individuals was also used as an independent variable. We fitted an additional model for potential fecundity in which wing size (length) was used as a covariate to test whether variation in potential fecundity was an indirect effect of wing size. Because of the unbalanced designs, we performed type‐III analysis of variance (Shaw & Mitchell‐Olds, 1993) for each of these models. We compared treatments using the Tukey's test (glht function in multcomp package). To improve interpretation of the results, effect sizes were calculated using partial eta‐squared (ƞ^2^ *p*, Cohen, 1973). Pairwise Spearman correlation was calculated between wings' length and wings' width. The temporal blocks did not modify the qualitative conclusions of our study when they were added as an independent variable; as a consequence, we decided to remove it from the models.

All analyses were performed with R version 4.0.2 (Team, 2020) with the “lme4” (Bates et al., 2009), “multcompView” (Graves et al., 2015), “effectsize” (Ben‐Shachar et al., 2021), and “car” (Fox & Weisberg, 2019) packages.

## 3 RESULTS

### 3.1 Oviposition assays: What range for larval density in a fruit?

Globally, 44.27% of the blueberries were infested (mean = 2.65 ± 1.75 per box). The number of eggs per fruit varied from 1 to 11 (mean = 1.34 ± 0.4 eggs per fruit). 31.77% of females oviposited more than one egg per fruit. In average, the total number of eggs (1–24 eggs, mean = 8.03 ± 6.34 eggs) did not vary between replicates (i.e., between females, χ^2^_1_ = 0.078, *p* = .78).

ZINB turned out to be the best model to explain the distribution of the number of eggs per fruit (AIC = 158.96), showing that the distribution of eggs was in aggregates with a greater number of noninfested fruits than expected under negative binomial distribution (z = 6.47, *p* < .001; Figure S3).

### 3.2 Immediate effects of larval density

#### 3.2.1 Effect of larval density and resource volume on adult emergence

**FIGURE 2:**
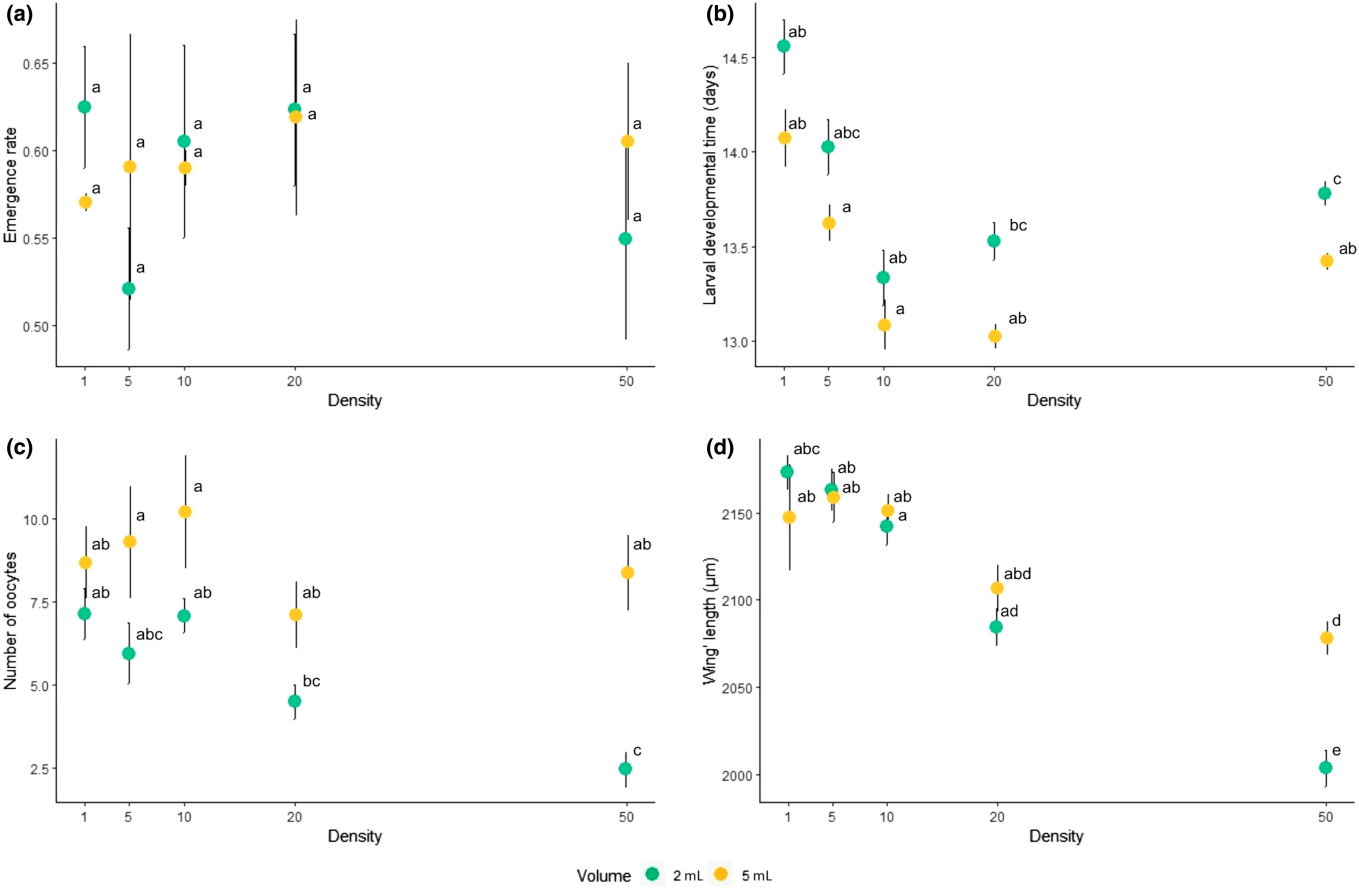
Effect of larval density and resource volume (2 (green) and 5 (yellow) mL of food medium) on immediate (a, b) and delayed (c, d) life‐history traits (mean ± SE) of *D. suzukii*. Panel (a) shows emergence rate. Panel (b) shows the developmental time for larvae to emerge in days. Panel (c) shows potential fecundity measured as the number of oocytes. Panel (d) shows wings' length. Different letters indicate statistically significant differences between treatments with alpha = 0.05 (post hoc Tukey test).

The average emergence rate was 0.59 [95% CI: 0.56–0.61]. It was not affected by larval density nor by the volume of food (χ^2^_1_ = 0.604, *p* = .43; χ^2^_1_ = 3.471, *p* = .06, respectively; Figure 2a). Although the interaction between these two variables was not significant (χ^2^_1_ = 3.541, *p* = .06), it had a large effect size (ƞ^2^ *p* = .17, [95% CI: 0.00–1.00]). Density square was also not significant (χ^2^_1_ = 0.743, *p* = .38).

### 3.3 Effect of larval density and resource volume on larval development time

The larval development time was affected by the volume of resource available for larval feeding (*F*_1,955_ = 22.889, *p* < .001), density (*F*_1,955_ = 99.736, *p* < .001), and sex (*F*_1,955_ = 22.559, *p* < .001). Interactions were not significant. In general, individuals that have grown in the lowest larval density (1 larva) took more days (14.31 ± 0.24) to develop compared with the other densities (difference of the mean development time: 0.83 ± 0.21 days; Figure 2b). Also, females took more time (13.87 ± 0.15 days) to develop than males (13.42 ± 0.14 days; Figure S4). Both volume and sex had a small effect size (ƞ^2^ *p* = .02, [95% CI: 0.01–1.00]), while density had a medium effect size (ƞ^2^ *p* = .09, [95% CI: 0.07–1.00]). Density square was also significant (*F*_1,955_ = 102.412, *p* < .001), which suggests a U‐shape relationship between density and development: The developmental time decreases with density, but after reaching a certain value (around 20 larvae) it increases (Figure 2b).

### 3.4 Delayed effects of larval density

#### 3.4.1 Effect of larval density and resource volume on female potential fecundity

Females that developed in low density (1 larva) presented more mature oocytes (mean = 7.91 ± 4.62) in the ovaries compared with those raised at high density (for 50 larvae, mean = 5.41 ± 4.31). There was a significant interaction between density and volume of resource (*F*_1,252_ = 11.819, *p* < .001; Figure 2c): When resources were limited (2 mL), females reared in the condition of 50 larvae had fewer oocytes than those that developed alone (z = −3.565, *p* < .05). On the contrary, the differences between densities were no longer significant when more resources were available (z = −0.213, *p* = 1.00). However, this interaction had a small effect size (ƞ^2^ *p* = .04, [95% CI: 0.01–1.00]). Density square was not significant (*F*_1,252_ = 0.318, *p* = .57). When we added the size (length of the wing) as a covariate in the model, the effect was significant (*F*_1,241_ = 17.56, *p* < .001), but the density (*F*_1,241_ = 0.95, *p* = .43) and the volume of resources (*F*_1,241_ = 0.45, *p* = .5) were then no longer significant.

#### 3.4.2 Effect of larval density and resource volume on wing length and width

As the length and the width of the wings were positively correlated (Spearman's *r*_255_ = .84, *p* < .001, Figure S5), we presented only results of the length (see the Supporting Information for width Table S3). There was a significant interaction between larval density and resource volume (*F*_1,252_ = 14.991, *p* < .001; Figure 2d) with a medium effect size (ƞ^2^ *p* = .06, [95% CI: 0.02–1.00]). This interaction was mainly due to differences between density 50 and the other densities (Table S2) where flies emerged with smaller wings (mean = 2040.9 ± 10.02 μm), and this effect was even more severe when there were less resources available (z = 4.094, *p* < .01). Density square was not significant (*F*_1,252_ = 2.4, *p* = .12).

## 4 DISCUSSION

Our study provides information on the range of eggs or larvae that can be found in fruits infested by *D. suzukii* and the potential effects of the larval density on major life‐history traits and their related trade‐offs in this pest.

In phytophagous species, where immatures develop within a finite volume of resources (e.g., fruits or seeds), mothers' oviposition strategy determines the fate of offspring and their fitness (Doak et al., 2006). Our results show that the oviposition strategy of *D. suzukii* females results in an aggregative distribution of eggs in fruits: Up to 11 eggs have been laid by one female in the same blueberry, with an average of more than 2.5 eggs per fruit. Thus, at least in our lab conditions (i.e., few available fruits), *D. suzukii* females do not avoid fruits already containing their own eggs. The grouping of the berries did not affect the oviposition decision of the females, which means that the females will not necessarily exploit the nearest substrate, but rather the aggregated eggs were found around the box. Some measures are available for this species in laboratory conditions and confirm the potential occurrence of high larval density per fruit (in raspberry juice agar: 10.33 ± 1.55 eggs, Elsensohn, Aly, et al., 2021). Although Elsensohn, Aly, et al. (2021); Elsensohn, Schal, and Burrack (2021) found that the oviposition rate decreased when the number of females per container increased, the density of eggs presents in the substrate did not affect the oviposition behavior of the females, but the presence of larvae and host marking did. However, females consistently laid more eggs on high‐quality diets (Elsensohn, Schal, & Burrack, 2021). This high density per fruit has been also described in the field (e.g., in raspberries 4.2 ± 1.3 immatures per berry (Burrack et al., 2013), 2.6 ± 0.8 in mulberries (Yu et al., 2013), 6.5 ± 0.7 in cultivated blueberries, and 2.5 ± 0.8 in wild blueberries (Rodriguez‐Saona et al., 2019)). Different authors suggest that larval crowding could be common in wild *Drosophila* populations, like in *D. melanogaster* (Atkinson, 1979; Roper et al., 1996), *D. aldrichi* and *D. buzzatii* (Krebs et al., 1992). Jaenike & James (1991) showed that this aggregation in the field might be due to the nonrandom choice of oviposition sites and oviposition in clutches in the case of *D. falleni*, *D. recens*, *D. putrida*, and *D. testacea*. Globally, these data suggest that, in conditions where fruits could be limiting (for instance in the start of the fruit‐growing season or in greenhouses cultures), high densities of immatures competing in a given fruit are likely.

Usually, larval density affects lifespan and other fitness traits such as development time like in *D. melanogaster* (Horváth & Kalinka, 2016). Here, we observe contrasted effects of density on larval life‐history traits. We do not find any change in preimaginal survival (from L1 to adult emergence) between densities and sexes. This is the case whatever the volume of resource (mimicking two sizes of fruits) while we expected an increase of the negative density‐dependent effects in the small resource volume. Since in *D. melanogaster*, survival can be affected by density due to the accumulation of feces in the environment and thus a deterioration in food quality (Joshi et al., 1998; Sarangi et al., 2016), we verified the number of bacterial colonies present in the medium in a preliminary experiment (for more information, see the Supporting Information). We found that, although the number of bacterial colonies in the food medium increases with larval density, this does not have any negative effect on preimaginal survival.

In contrast, the larval development time decreases with larval density, but after reaching a certain threshold (around 20 larvae) it increases again. A shorter development may be due to different factors. First, as observed in the flies *Bactrocera tryoni*, more intensive digestion of the food medium by larvae can lead to a facilitation in food intake and finally to a reduced developmental time (Morimoto et al., 2018). Second, a faster development can allow escaping competition and avoiding mortality driven by the risk of running out of resources before metamorphosis as found in *Scathophaga stercoraria* (Blanckenhorn, 1999). In our study, the U‐shape relationship between density and development time, and thus the increase after a threshold, may result from extra costs for food acquisition when developing at high density (in the case of *D. melanogaster* see Horváth & Kalinka, 2016; Miller, 1964; Nunney, 1996). For example, in *D. buzzatii* and *D. melanogaster*, a tolerance of waste products resulting from adaptation to crowded environments can have a cost in terms of nutrient extraction leading to extended larval development at high density (Betrán et al., 1998; Joshi & Mueller, 1996; Shakarad et al., 2005). However, in our experiments, larvae reared in the smallest volume of food (2 mL, i.e., the highest competition intensity) took more days to reach adult stage than those reared in 5 mL, regardless of larval density. This suggests that, in the face of increased intraspecific competition (due to a reduction in available resources), the larval strategy could compensate for the reduction of nutrient intake by increasing their development time. The same strategy was described in *D. melanogaster* and the moth *Cnaphalocrocis medinalis* (Mackay, 2001; Yang et al., 2015). Even so, the type of diet can alter the magnitude of these density effects, as Jaramillo et al. (2015) demonstrated in *D. suzukii*; they showed that the development time is shorter when the larvae fed on blueberries (natural host) compared with an artificial media. At last, we show that males emerged before females. A longer timing of maturation would allow females to reach a bigger size, which entails an advantage in their fecundity. This trade‐off has also been described on a wide range of insects (Honěk, 1993; Teder et al., 2021).

Density effects detected on immature stages had also consequences on the adult life‐history traits. One of the most remarkable effects of crowding during larval development is the decline of female fecundity. In our study, the females that experienced high larval densities have a lower number of mature oocytes present in their ovarioles. This negative impact is observed in numerous insects (Peckarsky & Cowan, 1991; Peters & Barbosa, 1977; Sato et al., 2004; Vamosi & Lesack, 2007). In *D. suzukii*, the decrease in the number of oocytes produced is positively correlated with a reduction of the wing size, a proxy of body size (see also for other insect species Honěk, 1993; Leather, 2018). However, further investigations are necessary to corroborate that the number of offspring, here the number of mature oocytes, decreases in response to the impact of larval density on the adult body size.

Our results show a phenotypic trade‐off between larval and adult life‐history traits. High larval density leads immatures to develop faster at the expense of adults' size and potential fecundity. Throughout the genus *Drosophila*, this trade‐off between juvenile developmental rate and adult viability is well‐described (Prasad et al., 2000). For example, *D. melanogaster* has an antagonistic pleiotropy between developmental rate and early‐ and late‐life survival (Chippindale et al., 2004). We assume that, at high density, female fitness is negatively affected by a reduction in flight ability, since we observed a reduction in wing size, and thus in the search for suitable oviposition sites, as well as by the risk of becoming egg‐limited due to reduced egg load in comparison with the availability of oviposition sites (Rosenheim, 2011). This reasoning implies a negative correlation between size and reproductive success in the field that is not always proven in insects (Ellers et al., 1998; West et al., 1996). An estimate of the impact of larval density on adult longevity would be relevant to an accurate estimation of density‐dependent effects on fitness. Although this study is conducted in laboratory conditions, we speculate that in their natural environment *D. suzukii* also face intraspecific competition with natural fruits and, therefore, it is likely that our results can be extrapolated beyond laboratory‐reared flies. However, similar experiments should be conducted with different lines of *D. suzukii* in the laboratory and/or under natural conditions to confirm the density dependence effects detected.

## 5 CONCLUSION

Females of *D. suzukii* laid their eggs in an aggregate distribution, which promotes crowding of the larvae. Contrary to expectations, preimaginal survival to adult emergence is not affected. However, larval development time shortened as density increases and resources become more limited. Furthermore, rearing at high larval densities negatively affects the fitness of adults which are smaller and have reduced potential fecundity, supporting the existence of trade‐off between larvae and adult life‐history traits.

## AUTHOR CONTRIBUTIONS

**Alicia Reyes‐Ramírez:** Formal analysis (equal); visualization (lead); writing – original draft (equal). **Zaïnab Belgaidi:** Methodology (lead). **Patricia Gibert:** Conceptualization (equal); writing – original draft (equal). **Thomas Pommier:** Methodology (equal); writing – original draft (equal). **Aurélie Siberchicot:** Formal analysis (equal). **Laurence Mouton:** Conceptualization (equal); project administration (equal); writing – original draft (equal). **Emmanuel Desouhant:** Conceptualization (lead); project administration (equal); writing – original draft (equal).

## CONFLICT OF INTEREST STATEMENT

The authors have declared that no competing interests exist.

## FUNDING INFORMATION

This project was funded by Agence Nationale de la Recherche (project ANR‐19‐CE32‐0010).

## Supporting information


Appendix S1.

## Data Availability

All data are available in Dryad data repository. doi: 10.5061/dryad.c59zw3rdw .
