# Supplementary material for: Larval density in the invasive Drosophila suzukii: Immediate and delayed effects on life‐history traits
Source: Ecol Evol. 2023 Aug 25;13(8):e10433. doi: 10.1002/ece3.10433 (PMC10450837; doi:10.1002/ece3.10433)
Supplement: Supplementary file 1 — Appendix S1. [file ECE3-13-e10433-s001.docx]

**Appendix**

*Abundance of microorganisms*

In order to test whether the larval density (and thus feeding and excretion) changes the amount of bacteria in the food medium, we inoculated medium from the vials where the larvae developed in. Using an inoculation loop, around 10µL of food medium were sampled under sterile conditions and diluted in 100µL of ultrapure water. These mixtures were then streaked on two solid growth media, LB (Lysogeny Broth amended with 5% Agar) and TSA (Tryptone Soy Agar), in 90 mm Petri dishes (8 boxes per modality and per medium, *i.e.* 160 Petri dishes in total). These two media are the most common media to cultivate aerobic bacteria. LB and TSA are not specific to any environment, thus providing nutrients for numerous strains. After sealing with parafilm to limit drought and cross-contaminations, the Petri dishes were incubated aerobically for 7 days at 37 °C in the dark. The counts were realized on ¼ randomly chosen part of the Petri dishes at the end of the incubation. Control Petri dishes, containing just the media, were incubated to evaluate potential contamination during incubation. All controls remained blank until the end of the experiment.

The effects of larval density on the number of microbial colonies per medium plate were analyzed by the means of a GLMM (Poisson distribution, log link). We also included the volume and the growth medium as independent variables, as well as all the double interactions; the tube where the aliquot was taken was included as a random factor. A Tukey test was used for comparisons between treatments.

The number of colonies increased with the density of larvae, and globally colonies were more numerous (TableS4) in the LB medium (316.32±118.82) as compared to the TSA medium (278.61±113.11). Likewise, the interaction between the volume of food and the growth medium was significant (TableS4), mainly due to a fewer number of colonies grown on TSA medium (112.28±39.53) compared to LB medium (116.46±38.26) in samples from 2 mL of resources.

Table S1. PCR primers and conditions used to check for the presence of *Wolbachia*

| **Primers** | **Primer sequences** | **Annealing temperature/ Product size** | **References** |
| --- | --- | --- | --- |
| **81F**  **2R** | 5’ – TGGTCCAATAAGTGATGAAGAAAC – 3’  5’- CAGCAATTTCAGGATTAG -3’ | 54°C / 290bp | Braig et al. 1998  Henri & Mouton 2012 |

DNA from single individuals was extracted using the NucleoSpin Tissue kit (Macherey-Nagel). *Wolbachia* detection was performed using *Wolbachia* specific primers that amplify a part of the *Wolbachia surface protein* gene. PCR reactions were performed in 10μL volumes containing 500nM primers, 1X Precision Melt Supermix (Biorad®) containing dNTPs, iTaq™ DNA polymerase, MgCl2, EvaGreen dye, stabilizers) and 2μL of DNA diluted to one tenth. Cycling conditions were 95°C for 2 min, then 30sec at 95°C, 30sec at 54°C and 30sec at 72°C for 40 cycles, followed by 30 sec at 95°C and 1min at 60°C (Biorad CFX96).

TableS2. Comparisons of the larval density on wings’ length according to the Tukey post-hoc test. Significant effects (p<0.05) are in bold.

| Density/Volume | estimate | z | p |
| --- | --- | --- | --- |
| D5.V2 – D1.V2 | -9.846 | -0.468 | 0.9 |
| D10.V2 – D1.V2 | -31.208 | -1.659 | 0.8 |
| D20.V2 – D1.V2 | -88.777 | -4.985 | **<0.001** |
| D50.V2 – D1.V2 | -169.499 | -9.517 | **<0.001** |
| D1.V5 – D1.V2 | -25.938 | -1.331 | 0.9 |
| D5.V5 – D1.V2 | -14.262 | -0.722 | 0.9 |
| D10.V5 – D1.V2 | -21.873 | -1.150 | 0.9 |
| D20.V5 – D1.V2 | -66.470 | -3.703 | **<0.01** |
| D50.V5 – D1.V2 | -95.203 | -5.164 | **<0.001** |
| D10.V2 – D5.V2 | -21.361 | -0.986 | 0.9 |
| D20.V2 – D5.V2 | -78.930 | -3.794 | **<0.01** |
| D50.V2 – D5.V2 | -159.653 | -7.674 | **<0.001** |
| D1.V5 – D5.V2 | -16.092 | -0.723 | 0.9 |
| D5.V5 – D5.V2 | -4.415 | -0.196 | 1 |
| D10.V5 – D5.V2 | -12.026 | -0.550 | 0.99 |
| D20.V5 – D5.V2 | -56.623 | -2.706 | 0.1 |
| D50.V5 – D5.V2 | -85.357 | -3.999 | **<0.01** |
| D20.V2 – D10.V2 | -57.569 | -3.107 | 0.06 |
| D50.V2 – D10.V2 | -138.291 | -7.463 | **<0.001** |
| D1.V5 – D10.V2 | 5.270 | 0.262 | 1 |
| D5.V5 – D10.V2 | 16.946 | 0.830 | 0.9 |
| D10.V5 – D10.V2 | 9.335 | 0.474 | 0.9 |
| D20.V5 – D10.V2 | -35.262 | -1.889 | 0.6 |
| D50.V5 – D10.V2 | -63.995 | -3.345 | **<0.05** |
| D50.V2 – D20.V2 | -80.722 | -4.610 | **<0.001** |
| D1.V5 – D20.V2 | 62.839 | 3.270 | **<0.05** |
| D5.V5 – D20.V2 | 74.515 | 3.825 | **<0.01** |
| D10.V5 – D20.V2 | 66.904 | 3.569 | **<0.05** |
| D20.V5 – D20.V2 | 22.307 | 1.263 | 0.9 |
| D50.V5 – D20.V2 | -6.426 | -0.354 | 1 |
| D1.V5 – D50.V2 | 143.561 | 7.470 | **<0.001** |
| D5.V5 – D50.V2 | 155.237 | 7.968 | **<0.001** |
| D10.V5 – D50.V2 | 147.626 | 7.876 | **<0.001** |
| D20.V5 – D50.V2 | 103.029 | 5.836 | **<0.001** |
| D50.V5 – D50.V2 | 74.296 | 4.094 | **<0.001** |
| D5.V5 – D1.V5 | 11.676 | 0.555 | 0.9 |
| D10.V5 – D1.V5 | 4.065 | 0.200 | 1 |
| D20.V5 – D1.V5 | -40.532 | -2.095 | 0.5 |
| D50.V5 – D1.V5 | -69.265 | -3.498 | **<0.05** |
| D10.V5 – D5.V5 | -7.611 | -0.369 | 1 |
| D20.V5 – D5.V5 | -52.208 | -2.662 | 0.1 |
| D50.V5 – D5.V5 | -80.942 | -4.035 | **<0.01** |
| D20.V5 – D10.V5 | -44.597 | -2.362 | 0.3 |
| D50.V5 – D10.V5 | -73.330 | -3.792 | **<0.01** |
| D50.V5 – D20.V5 | -28.733 | -1.571 | 0.8 |

TableS3. Comparisons of the larval density on wings’ width according to the Tukey post-hoc test. Significant effects (p<0.05) are in bold.

| Density/Volume | estimate | z | p |
| --- | --- | --- | --- |
| D5.V2 – D1.V2 | -15.605 | -1.197 | 0.9 |
| D10.V2 – D1.V2 | -30.660 | -2.632 | 0.2 |
| D20.V2 – D1.V2 | -61.618 | -5.589 | **<0.01** |
| D50.V2 – D1.V2 | -110.652 | -10.036 | **<0.01** |
| D1.V5 – D1.V2 | -14.309 | -1.186 | 0.9 |
| D5.V5 – D1.V2 | -18.613 | -1.522 | 0.8 |
| D10.V5 – D1.V2 | -22.322 | -1.895 | 0.6 |
| D20.V5 – D1.V2 | -47.464 | -4.271 | **<0.01** |
| D50.V5 – D1.V2 | -57.856 | -5.069 | **<0.01** |
| D10.V2 – D5.V2 | -15.054 | -1.122 | 0.9 |
| D20.V2 – D5.V2 | -46.013 | -3.572 | **<0.05** |
| D50.V2 – D5.V2 | -95.047 | -7.380 | **<0.001** |
| D1.V5 – D5.V2 | 1.296 | 0.094 | 1 |
| D5.V5 – D5.V2 | -3.008 | -0.216 | 1 |
| D10.V5 – D5.V2 | -6.717 | -0.497 | 1 |
| D20.V5 – D5.V2 | -31.859 | -2.459 | 0.2 |
| D50.V5 – D5.V2 | -42.250 | -3.198 | **<0.05** |
| D20.V2 – D10.V2 | -30.958 | -2.699 | 0.1 |
| D50.V2 – D10.V2 | -79.993 | -6.973 | **<0.001** |
| D1.V5 – D10.V2 | 16.350 | 1.311 | 0.9 |
| D5.V5 – D10.V2 | 12.046 | 0.954 | 0.9 |
| D10.V5 – D10.V2 | 8.337 | 0.684 | 0.9 |
| D20.V5 – D10.V2 | -16.804 | -1.454 | 0.9 |
| D50.V5 – D10.V2 | -27.196 | -2.296 | 0.3 |
| D50.V2 – D20.V2 | -49.035 | -4.524 | **<0.001** |
| D1.V5 – D20.V2 | 47.309 | 3.977 | **<0.01** |
| D5.V5 – D20.V2 | 43.005 | 3.565 | **<0.05** |
| D10.V5 – D20.V2 | 39.295 | 3.387 | **<0.05** |
| D20.V5 – D20.V2 | 14.154 | 1.295 | 0.9 |
| D50.V5 – D20.V2 | 3.762 | 0.335 | 1 |
| D1.V5 – D50.V2 | 96.343 | 8.098 | **<0.001** |
| D5.V5 – D50.V2 | 92.039 | 7.631 | **<0.001** |
| D10.V5 – D50.V2 | 88.330 | 7.612 | **<0.001** |
| D20.V5 – D50.V2 | 63.188 | 5.781 | **<0.001** |
| D50.V5 – D50.V2 | 52.797 | 4.700 | **<0.001** |
| D5.V5 – D1.V5 | -4.304 | -0.331 | 1 |
| D10.V5 – D1.V5 | -8.013 | -0.636 | 0.9 |
| D20.V5 – D1.V5 | -33.155 | -2.768 | 0.1 |
| D50.V5 – D1.V5 | -43.547 | -3.553 | **<0.05** |
| D10.V5 – D5.V5 | -3.709 | -0.291 | 1 |
| D20.V5 – D5.V5 | -28.851 | -2.376 | 0.3 |
| D50.V5 – D5.V5 | -39.242 | -3.160 | 0.05 |
| D20.V5 – D10.V5 | -25.142 | -2.151 | 0.4 |
| D50.V5 – D10.V5 | -35.533 | -2.968 | 0.08 |
| D50.V5 – D20.V5 | -10.392 | -0.918 | 0.9 |

TableS4. Results of the GLMM evaluating the larval density and resource volume on microbial colony counts present in the medium where *D. suzukii* larvae develop. Significant effects (p<0.05) are in bold.

|  | df | χ^2^ | p |
| --- | --- | --- | --- |
| Density | 4 | 19.87 | **<0.001** |
| Volume | 1 | 0.044 | 0.83 |
| Medium | 1 | 25.739 | **<0.001** |
| Density × Volume | 4 | 6.429 | 0.17 |
| Density × Medium | 4 | 112.857 | **<0.001** |
| Volume × Medium | 1 | 21.323 | **<0.001** |


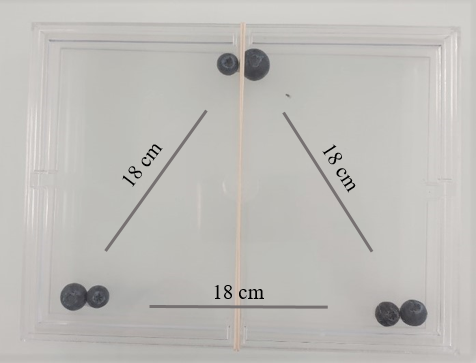


**First**

**Second**

**Third**

FigureS1. Experimental set up for the oviposition assays on blueberries. Three groups of 2 blueberries were placed at equal distance from each other in a box (23.8cm x 17.8cm x 2cm).


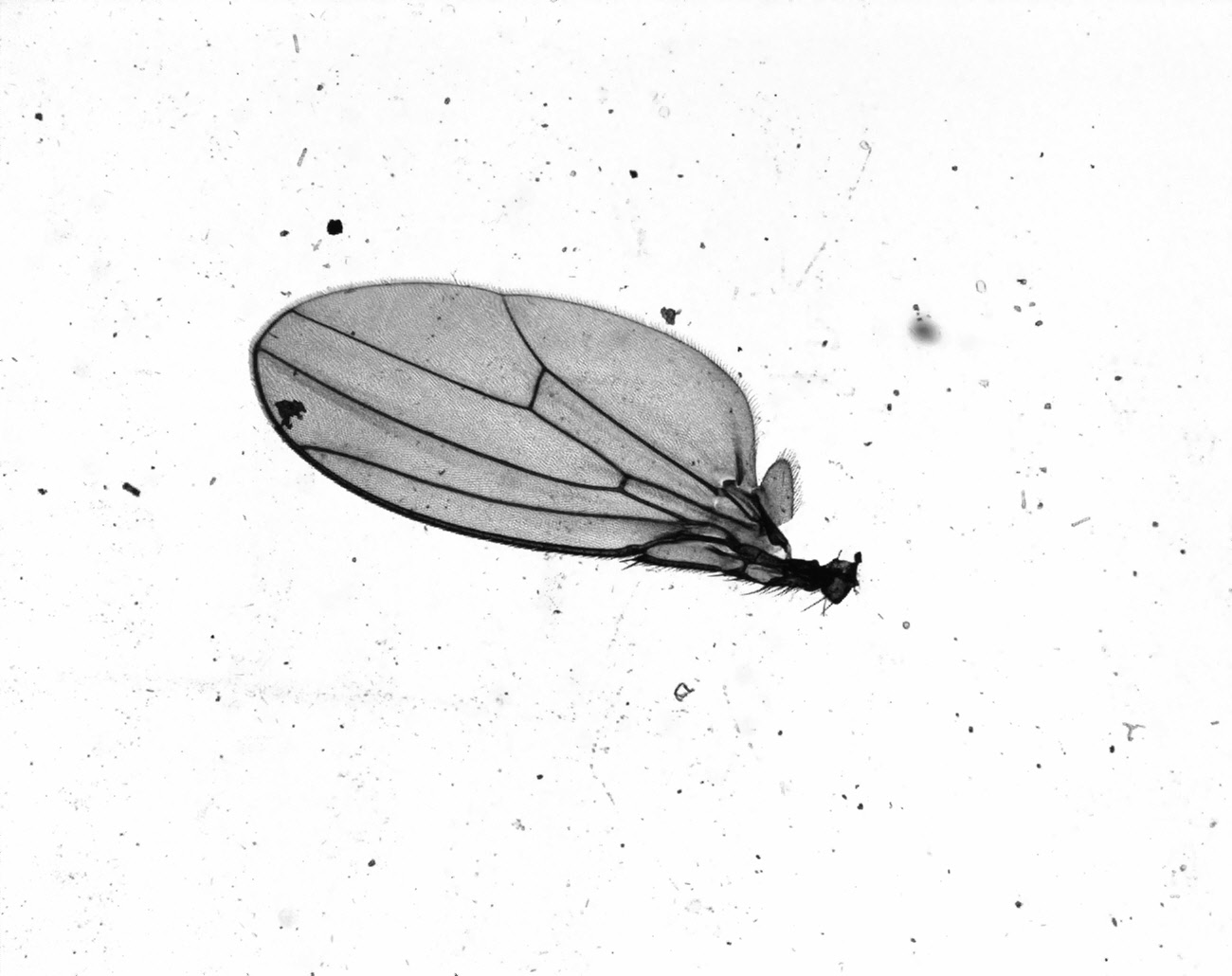


FigureS2. Measures done for the wing size. The two red lines indicated the measures done.


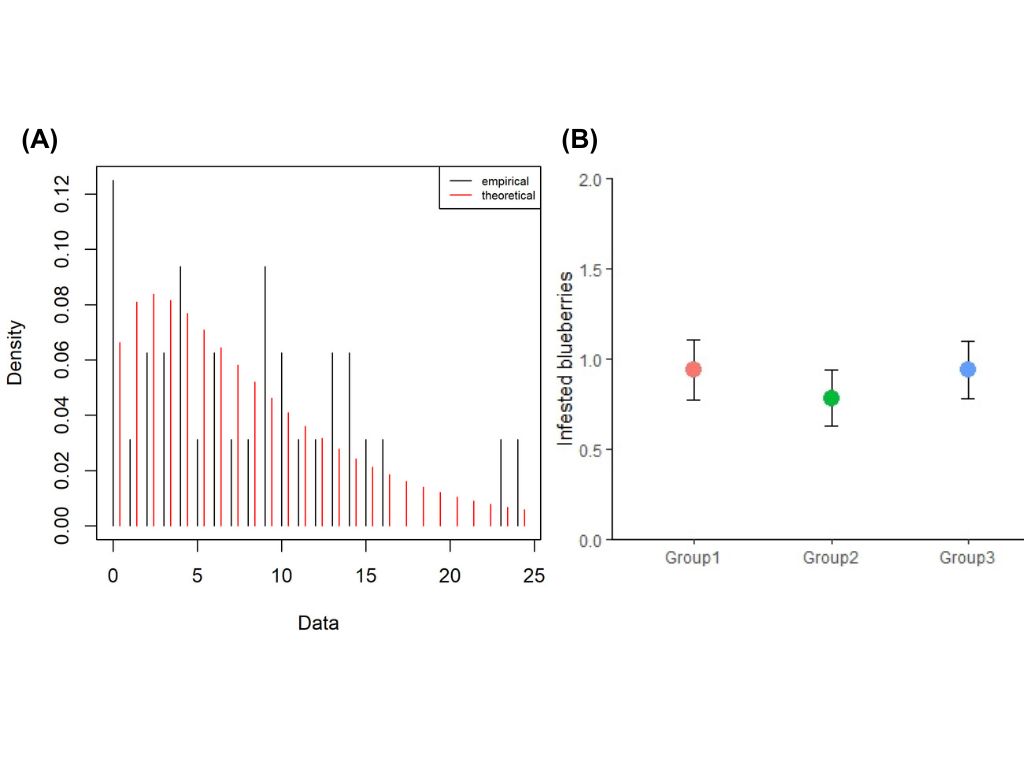


FigureS3. Comparison between the empirical and theoretical (zero-inflated negative binomial regression, ZINB) distribution of *D. suzukii* eggs deposited per infested fruit (A). Number of infested blueberries (mean ± SE) in each group in the boxes of the oviposition experiment (B). We did not detect any difference in the number of infested blueberries per group (χ^2^_1_=0.592, p=0.743) or per box (χ^2^_1_=0.826, p=0.363).


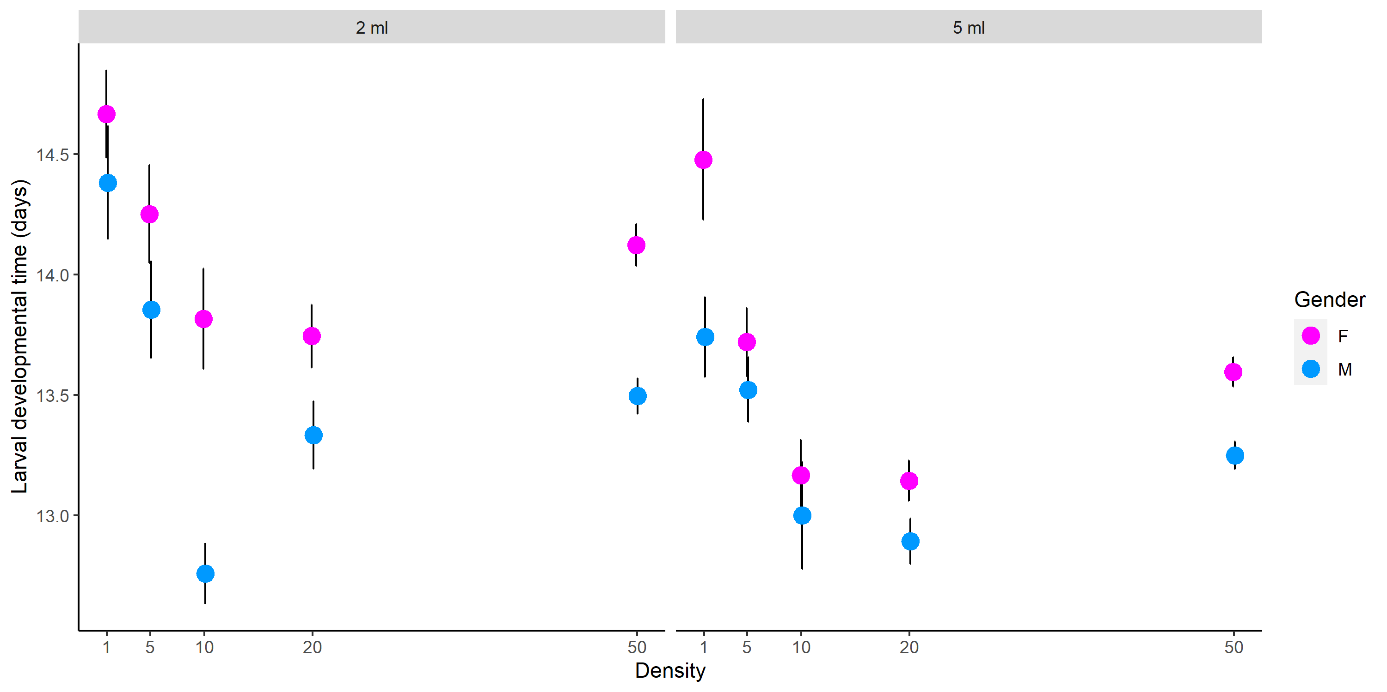


FigureS4. Effect of larval density and resource volume (2 and 5 mL of food medium) on larval developmental time (mean ± SE) between females (pink) and males (blue) of *D. suzukii*.


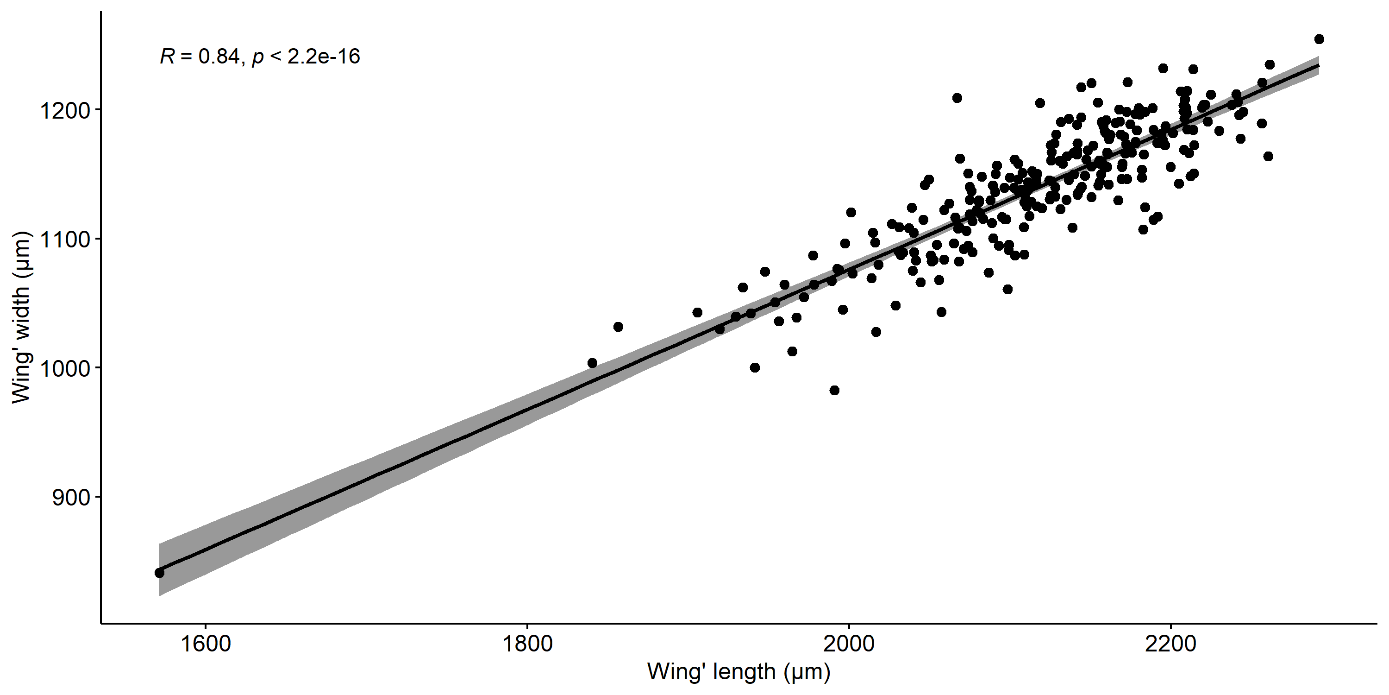


FigureS5. Spearman’ correlation coefficient (R) and linear regression line between wings’ length and wings’ width means. Each point corresponds to one individual (n=257).
